# A commentary on “Application and current challenges of neural stem cells transplantation in clinical trials of spinal cord injury”

**DOI:** 10.1097/JS9.0000000000002935

**Published:** 2025-07-04

**Authors:** Bowen Sang, Xiaoyong Qin, Mingdan Zhu

**Affiliations:** a Heilongjiang University of Chinese Medicine, Harbin, Heilongjiang; b Department of Acupuncture and Moxibustion, The First Affiliated Hospital of Heilongjiang University of Chinese Medicine, Harbin, Heilongjiang; c Department of Neurology, The 969th Hospital of the Joint Logistics Support Force of P.L.A, Huhhot, Inner Mongolia, China; d Liaoning Academy of Traditional Chinese Medicine Sciences, Liaoning University of Traditional Chinese Medicine, Shenyang, Liaoning, China

*Dear Editor*,

We read with great interest the recent article titled “Application and current challenges of neural stem cells transplantation in clinical trials of spinal cord injury”^[1]^ published in the *International Journal of Surgery*. The goal of this study is to provide valuable information for the future use of NSCs in SCI by thoroughly discussing their usage in clinical trials as well as the issues and difficulties that now exist. However, a number of significant issues need to be addressed in future research.

First, although the paper gives a thorough summary of how neural stem cells (NSCs) are used to treat spinal cord injuries (SCI), it does not go into great length about how clinical studies are designed^[2]^. For instance, most clinical trials have small sample sizes (ranging from 4 to 12 patients) and lack randomized controlled designs, which can lead to biased results. Additionally, the follow-up periods are relatively short (up to 6 years), making it challenging to assess long-term efficacy and safety. The study also fails to clearly define inclusion and exclusion criteria, potentially affecting the generalizability of the findings. Future research should increase the sample size, extend the follow-up period, and adopt a multicenter randomized controlled trial (RCT) design to enhance the evidence level.

Second, the article lists a number of transplanting techniques without weighing the benefits and drawbacks of each. For example, intravenous injection can lead to cell dispersion, while local injection may increase surgical risks. Moreover, the optimal transplantation dose (ranging from 15 M to 40 M cells) has not been clearly defined, and there is a lack of studies on dose-effect relationships. Future research should focus on determining the best transplantation route and dose through preclinical experiments, and exploring non-invasive delivery methods, such as exosome carriers^[3]^.

Third, there is a lack of objective neurophysiological or imaging evidence, and the evaluation standards (such the ISNCSCI score) are not standardized, even if the paper notes that some patients have demonstrated improvements in motor or sensory abilities. For example, the correlation between T2 signal changes observed on MRI and functional recovery is unclear. It is recommended that future evaluations incorporate multimodal assessments (such as fMRI and motor evoked potentials) and patient-reported outcomes (PROs) to comprehensively measure treatment efficacy.

This review systematically summarizes the application of NSCs in SCI treatment, but there are deficiencies in research design, cell preparation, transplantation methods, functional evaluation, safety and combination therapy. In the future, clinical transformation should be promoted through large sample RCT, standardized cell production, multimodal evaluation and interdisciplinary cooperation. All of this was noted in relation to the TITAN guideline, which calls for transparency in the reporting of artificial intelligence^[4]^.

## Data Availability

Not applicable.

## References

[1] Zhang WJ Zhang J. Application and current challenges of neural stem cells transplantation in clinical trials of spinal cord injury. Int J Surg 2025:10–97. 10.1097/JS9.0000000000002548 40465790

[2] Khorasanizadeh M Yousefifard M Eskian M. Neurological recovery following traumatic spinal cord injury: a systematic review and meta-analysis. J Neurosurg Spine 2019;30:683–99. 30771786 10.3171/2018.10.SPINE18802

[3] Uchida N Chen K Dohse M. Human neural stem cells induce functional myelination in mice with severe dysmyelination. Sci Trans Med 2012;4:155ra36. 10.1126/scitranslmed.3004371 PMC3864816 23052293

[4] Agha RA Mathew G Rashid R. Transparency In The reporting of Artificial INtelligence – the TITAN guideline. Prem J Sci 2025;10:100082.

